# Endoscopic nodal staging in oligometastatic non-small cell lung cancer (NSCLC) being treated with stereotactic ablative radiotherapy (ENDO-SABR)

**DOI:** 10.1186/s12885-022-09563-8

**Published:** 2022-04-28

**Authors:** Inderdeep Dhaliwal, Shayan Kassirian, Michael A. Mitchell, Mehdi Qiabi, Andrew Warner, Alexander V. Louie, Harvey H. Wong, Christine M. McDonald, Jason Rajchgot, David A. Palma

**Affiliations:** 1 Division of Respirology, Department of Medicine, London Health Sciences Centre, Western University, London, Ontario Canada; 2 Division of Thoracic Surgery, Department of Surgery, London Health Sciences Centre, Western University, London, Ontario Canada; 3 Division of Radiation Oncology, Department of Oncology, London Health Sciences Centre, Western University, London, Ontario Canada; 4 Division of Radiation Oncology, Department of Oncology, Sunnybrook Health Sciences Centre, University of Toronto, Toronto, Ontario Canada; 5 Division of Respirology, Department of Medicine, Sunnybrook Health Sciences Centre, University of Toronto, Toronto, Ontario Canada

**Keywords:** Stereotactic ablative radiotherapy (SABR), Non-small cell lung cancer (NSCLC), Endobronchial ultrasound (EBUS), Endoscopic ultrasound (EUS), Oligometastatic disease

## Abstract

**Background:**

Research in treatment of non-small cell lung cancer (NSCLC) has shown promising results with stereotactic ablative radiotherapy (SABR) of oligometastatic disease, wherein distant disease may be limited to one or a few distant organs by host factors. Traditionally, PET/CT has been used in detecting metastatic disease and avoiding futile surgical intervention, however, sensitivity and specificity is limited to only 81 and 79%, respectively. Mediastinal staging still identifies occult nodal disease in up to 20% of NSCLC patients initially thought to be operative candidates. Endobronchial ultrasound and transbronchial needle aspiration (EBUS-TBNA) is a minimally invasive tool for the staging and diagnosis of thoracic malignancy. When EBUS is combined with endoscopic ultrasound using the same bronchoscope (EUS-B), the diagnostic sensitivity and negative predictive value increase to 84 and 97%, respectively. Endoscopic staging in patients with advanced disease has never been studied, but may inform treatment if a curative SABR approach is being taken.

**Methods:**

This is a multi-centre, prospective, cohort study with two-stage design. In the first stage, 10 patients with oligometastatic NSCLC (lung tumour ± hilar/mediastinal lymphadenopathy) with up to 5 synchronous metastases will be enrolled An additional 19 patients will be enrolled in the second stage if rate of treatment change is greater than 10% in the first stage. Patients will be subject to EBUS or combined modality EBUS/EUS-B to assess bilateral lymph node stations using a N3 to N2 to N1 progression. Primary endpoint is defined as the rate of change to treatment plan including change from SABR to conventional dose radiation, change in mediastinal radiation field, and change from curative to palliative intent treatment.

**Discussion:**

If a curative approach with SABR for oligometastatic disease is being explored, invasive mediastinal staging may guide treatment and prognosis. This study will provide insight into the use of endoscopic mediastinal staging in determining changes in treatment plan of NSCLC. Results will inform the design of future phase II trials.

**Trial registration:**

Clinicaltrials.gov identifier NCT04852588. Date of registration: April 19, 2021. Protocol version: 1.1 on December 9, 2021.

## Background

Historically, the treatment of advanced non-small cell lung cancer (NSCLC) has focused on minimizing symptoms, and prolonging life [1]. The oligometastatic paradigm of cancer argues that distant disease is not widespread, but rather constrained by host anatomical and physiological factors, into one or a few distant organs [2, 3]. Recently, a phase II study evaluating aggressive treatment with stereotactic ablative radiotherapy (SABR) of oligometastatic disease of any cell type, has been associated with an improvement in overall survival compared to standard of care [4]. Phase III studies are currently enrolling patients to validate the results of this promising study.

Regional or distant metastasis in NSCLC are non-invasively assessed by computed tomography (CT), positron emission tomography (PET), integrated PET/CT, magnetic resonance imaging (MRI) and radionucleotide bone scans [5]. PET/CT has been particularly useful in detecting occult metastases and avoiding futile surgical resection [6]. However, a large meta-analysis of integrated PET/CT for mediastinal nodal involvement in NSCLC reported sensitivity and specificity of only 81 and 79%, respectively [7]. Despite widespread reliance on PET/CT, mediastinal staging still identifies occult nodal disease in up to 20% of NSCLC patients who are operative candidates [8, 9].

Endobronchial ultrasound and transbronchial needle aspiration (EBUS-TBNA) for invasive mediastinal staging is a powerful and minimally invasive tool used to stage and diagnose thoracic malignancies [5, 8–10]. When EBUS is combined with endoscopic ultrasound (EUS) using the same bronchoscope (EUS-B), the diagnostic sensitivity and negative predictive value increase to 84 and 97%, respectively [11].

Treatment paradigms in the past did not justify invasive mediastinal staging for patients with advanced disease, therefore the presence of occult mediastinal metastasis has never been elucidated. However, if a curative approach for oligometastatic disease is being explored, invasive mediastinal staging may inform treatment planning and guide prognosis.

## Methods/design

The purpose of this study is to evaluate the impact of invasive mediastinal staging for patients undergoing curative treatment for oligometastatic NSCLC.

This study is a prospective cohort study with two-stage design. Ten patients will be enrolled initially and analyzed in the first stage. If the rate of treatment change is greater than 10%, 19 additional patients will be enrolled to complete the second stage of the study. The study will be conducted at two academic centers in Ontario, Canada. (London Health Sciences Centre, Sunnybrook Health Sciences Centre). Data will be collected by investigators and clinical trial coordinators into a secured, prospective RedCap (The Vanderbilt University) database.

Ethics approval was obtained from the Ontario Cancer Research Ethics Board (#3575).

### Objectives

#### Primary endpoints

1. To determine the proportion of patients with changes to treatment intent and/or plan, defined as:
  - Change from SABR to conventional dose radiation
  - Change in mediastinal radiation field
  - Change from curative intent to palliative intent treatment

#### Secondary endpoints

1. To determine the proportion of patients with occult mediastinal metastasis
2. To determine the sensitivity and specificity of radiologic staging of hilar/mediastinal lymph nodes
3. To identify the total number of lymph nodes sampled per procedure
4. To identify the nodal stations sampled
5. To determine the proportion of patients with complications from endoscopic staging

### Patient selection

#### Inclusion criteria

1. Patients with oligometastatic NSCLC, including a primary lung tumour (± hilar/mediastinal adenopathy) with up to 5 synchronous metastases
2. Radical treatment intent to all sites is recommended at multi-disciplinary tumour board or by discussion by medical oncologist, radiation oncologist and/or surgeon.
3. Age 18 years or older
4. Good performance status (Eastern Cooperative Oncology Group [ECOG] 0–1) with life-expectancy of at least 6 months as determined by enrolling physician
5. Patient has undergone staging investigations less than 3 months prior to registration.
  - PET/CT and MRI brain (CT brain with contrast if contraindication to MRI) OR
  - CT chest/abdomen, radionucleotide bone scan and MRI of brain (CT brain with contrast if contraindication to MRI)
6. Pathologic confirmation of NSCLC

#### Exclusion criteria

1. Contraindication to EBUS and/or EUS
2. Unable to provide consent for EBUS/EUS
3. Contraindication to chest radiotherapy
4. Pregnant or lactating women

### Intervention

#### Pre-treatment evaluation

- Staging within 12 weeks prior to registration with either:
  - Contrast-enhanced CT of the chest and abdomen, plus bone scan, plus MRI brain or
  - Whole body PET/CT and MRI brain
    (note: CT brain allowed when MRI contraindicated)
- Histological confirmation of NSCLC
- Pregnancy test for women of child-bearing potential
- Signed Informed Consent prior to any study-specific activities

#### Endoscopic procedure

All procedures will be performed by one of the study investigators. Procedures will be performed in an endoscopy setting with moderate conscious sedation, without the use of general anesthesia. EBUS-TBNA will be used to assess bilateral lymph nodal stations in the following areas: 2, 3P, 4, 5, 7, 10, and 11. EBUS may be combined with EUS-B to enhance accessibility to stations 4 L, 7, 5, 8, and 9. The use of EUS-B is not mandatory. EBUS will always be performed initially to reduce risk of airway contamination. Nodal assessment will be done systematically using a N3 to N2 to N1 progression, and any lymph node > 5 mm in short axis or with malignant features (one or more of: round shape, hypoechoic, loss of central hilar structures, central necrosis) will be sampled a minimum of 3 times. A new needle will be obtained upon stage migration to avoid contamination. Rapid onsite cytological evaluation (ROSE) will not be used for assessing samples. All specimens will be placed into formalin fixative followed by a needle-rinse into cytolyte and formally reviewed by a cytopathologist.

#### Radiation therapy

Radiation treatment intent and dosage will be initially planned based on clinical imaging, prescribed by the treating radiation oncologist and/or based on discussion at a multi-disciplinary cancer conference. Following endoscopic staging, any changes to radiation treatment intent or dosage will be recorded.

### Adverse events

#### Definitions

Adverse Event (AE) or reaction is any unfavorable and unintended sign (including an abnormal laboratory finding), symptom, or disease temporally associated with the use of a medical treatment or procedure that may or may not be considered related to the medical treatment or procedure.

Serious Adverse Event (SAE) or reaction as defined in the ICH Guideline: Clinical Safety Data Management: Definitions and Standards for Expedited Reporting, E2A Section IIB includes any untoward medical occurrence that at any dose:

- Results in death
- Is life-threatening (refers to an event in which the patient was at risk of death at the time of the event; it does not refer to an event which hypothetically might have caused death if it were more severe.)
- Results in persistent or significant disability/incapacity.
- Requires in-patient hospitalization or prolongation of existing hospitalization.
- Is a congenital anomaly/birth defect.

Important medical events that may not be immediately life-threatening or result in death or hospitalization may be considered a serious adverse event, when, based upon medical and scientific judgment, they may jeopardize the patient or may require intervention to prevent one of the other outcomes listed in the definition above.

Unexpected adverse reaction is one that the nature and severity is not consistent with the applicable product information (e.g., Investigator’s Brochure or Product Monograph, described in the REB/IRB approved research protocol or informed consent document), or occurs with more than expected frequency.

#### Causality (attribution)

An adverse event or reaction is considered related to the research intervention if there is a reasonable possibility that the reaction or event may have been caused by the research intervention (i.e. a causal relationship between the reaction and the research intervention cannot be ruled out by the investigator(s)). The relationship of an AE to the study treatment (causality) will be described using the following definitions:

Unrelated: Any adverse event for which there is evidence that an alternative etiology exists or for which no timely relationship exists to the administration of the study treatment and the adverse event does not follow any previously documented pattern. The adverse event, after careful consideration by the investigator, is clearly and incontrovertibly due to causes other than the intervention.

Unlikely: Any adverse event for which the time relationship between the study treatment and the event suggests that a causal relationship is unlikely and/or the event is more likely due to the subject’s clinical condition or other therapies concomitantly administered to the subject.

Possible: Any adverse event occurring in a timely manner after the administration of the study treatment that follows a known pattern to the intervention and for which no other explanation is known. The adverse event, after careful consideration by the investigator, is considered to be unlikely related but cannot be ruled out with certainty.

Probable: Any adverse event occurring in a timely manner after the administration of the study treatment that follows a known pattern to the intervention and for which no other explanation is known. The adverse event, after careful consideration by the investigator, is believed with a high degree of certainty to be related to the intervention.

Definitely Related: Any adverse event occurring within a timely manner after administration of the study treatment that is a known sequela of the intervention and follows a previously documented pattern but for which no other explanation is known. The adverse event is believed by the investigator to be incontrovertibly related to the intervention.

#### Severity

The severity of adverse events will be evaluated using the Common Terminology Criteria for Adverse Events (CTCAE) v5.0 grading scale (see (http://ctep.cancer.gov).

Grade 1: Mild.

Grade 2: Moderate.

Grade 3: Severe.

Grade 4: Life-threatening or disabling.

Grade 5: Death.

Note: The term “severe” is a measure of intensity: thus a severe adverse event is not necessarily serious. For example, nausea of several hours’ duration may be rated as severe, but may not be clinically serious.

#### Immediately reportable adverse events

Any grade 4 or 5 adverse reaction that is definitely, probably, or possibly the result of protocol treatment must be verbally reported to the Principal Investigator and Central Office within 24 h of discovery.

All serious, unexpected adverse events or reactions regardless of causality must be reported to the Central Office and the Principal Investigator within 24 h of discovery. All unexpected SAEs must be reported to the local research ethics board according to local policies.

#### Subject discontinuation/withdrawal

Subjects may voluntarily discontinue participation in the study at any time. If a subject is removed from the study, the clinical and laboratory evaluations that would have been performed at the end of the study should be obtained. If a subject is removed because of an adverse event, they should remain under medical observation as long as deemed appropriate by the treating physician.

#### Follow-up evaluation

No study-specific follow-up visits are required for study participants, clinic visits will be standard of care.

### Statistical considerations

#### Sample size

Simon’s two-stage design will be used to determine the proportion of patients with treatment changes following EBUS/EUS-B staging. The null hypothesis that the true response rate is 10% will be tested against a one-sided alternative. In the first stage, 10 patients will be accrued. If there are 1 or fewer responses (treatment changes) in these 10 patients, the study will be stopped. Otherwise, 19 additional patients will be accrued for a total of 29. The null hypothesis will be rejected if 6 or more responses (treatment changes) are observed in 29 patients. This design yields an alpha of 0.05 and power of 80% when/if the true treatment change rate is 30%.

#### Statistical analysis

Descriptive statistics will be generated for baseline patient, tumour and treatment characteristics for all patients enrolled in either the first stage (*n* = 10) or second stage (*n* = 19) of the Simon’s two-stage design. The primary endpoint of this study will be to determine the proportion of patients with changes to treatment intent and/or plan, which will be reported with a 95% confidence interval and compared against the null hypothesis of no changes (0%) using the binomial proportion test. The secondary endpoints of this study will include determining the proportion of patients with occult mediastinal metastasis, which will also be reported with a 95% confidence interval. The sensitivity, specificity and concordance (using univariable logistic regression) measuring the association between radiologic staging of hilar/mediastinal lymph nodes (predictor variable) and changes in treatment intent and/or plan (dependent variable) will be calculated. The total number of lymph nodes sampled per procedure and proportion sampled for each nodal station will be calculated for each patient. The proportion of patients with complications of endoscopic procedure will also be reported. All statistical analyses will be performed using SAS version 9.4 software (SAS Institute, Cary, NC, USA) with two-sided statistical testing at the 0.05 significance level.

### Ethical considerations

#### Research ethics board

The protocol (and any amendments), the informed consent form, and any other written information to be given to subjects has been reviewed and approved by the Ontario Cancer Research Ethics Board, operating in accordance with the current federal regulations. The Clinical Trials Ontario Project ID is 3575. Any institution opening this study will obtain local IRB/REB approval prior to local initiation.

#### Informed consent

The written informed consent form is to be provided to potential study subjects and should be approved by the local IRB/REB and adhere to principles of the International Council for Harmonisation Guidelines for Good Clinical Practice (ICH GCP), which have their origins in the Declaration of Helsinki. The study consent form can be found in additional forms. The investigator is responsible for obtaining written informed consent from each subject, or if the subject is unable to provide informed consent, the subject’s legally acceptable representative, prior to beginning any study procedures and treatment(s). The investigator should inform the subject, or the subject’s legally acceptable representative, of all aspects of the study, including the potential risks and benefits involved. The subject should be given ample time and opportunity to ask questions prior to deciding about participating in the study and be informed that participation in the study is voluntary and that they are completely free to refuse to enter the study or to withdraw from it at any time, for any reason. The informed consent must be signed and dated by the subject, or the subject’s legally acceptable representative, and by the person who conducted the informed consent discussion. A copy of the signed and dated written informed consent form should be given to the subject or the subject’s legally acceptable representative. The process of obtaining informed consent should be documented in the patient source documents.

#### Confidentiality

The names and personal information of study participants will be held in strict confidence. All study records will only identify the subject by initials and the assigned study identification number. The study coordinator will maintain a confidential subject identification list (Master List) during the course of the study. Access to confidential information (i.e., source documents and patient records) is only permitted for direct subject management and for those involved in monitoring the conduct of the study. The subject’s name will not be used in any public report of the study.

### Authorship

Upon completion of this project the results will be published in a peer-reviewed journal and/or medical conference(s).

Final decisions on authorship will be made by the primary and senior authors (ID and DAP). The authorship group otherwise will represent each centre accruing patients provided co-authors contribute as outlined by ICMJE.

## Discussion

Oligometastatic disease, particularly in NSCLC, is a relatively novel concept with emerging research. Therapeutic options may differ in oligometastatic disease, such as SABR to all sites of disease, and may confer mortality benefit [4]. To accompany evolving therapeutics, diagnostic tools should improve to more accurately distinguish oligometastatic disease. The use of endoscopic staging as a minimally invasive tool to improve mediastinal staging sensitivity would in turn allow for more accurate and personalized therapeutic options.

Synergistic to the objectives of our study, a prediction model incorporating demographics, CT/PET results, and primary tumor characteristics can suggest nodal stage in NSCLC patients being considered for SABR, confirmed ultimately by EBUS [12]. This model may assist in determining a population that would benefit most from EBUS nodal staging. In addition, EBUS in staging NSCLC has already been determined to be more cost effective compared to other invasive modalities [13] – however its cost effectiveness with respect to oligometastatic disease with the availability of SABR has yet to be determined.

Limitations of our study include the inability to accurately assess risk of complications with a small sample size. To ensure that the invasive endoscopic procedure would not be futile, we chose a two-stage design, and will discontinue enrollment if there is < 10% treatment change in the first stage of enrollment. Furthermore, the clinical efficacy of this approach cannot be assessed by this study, and will need to be assessed in larger, prospective study. An economic evaluation is also not performed.

This two-stage cohort study aims to personalize NSCLC treatment approach by using endoscopic staging to match them to a more accurate treatment modality, thereby sparing them and the health care system undue burden. Data from this study may be used to inform phase II investigations.

## Data Availability

Upon completion of the clinical trial, anonymized data from the study will be published into a manuscript submitted for peer reviewed scientific journal publication and/or scientific conference presentation.

## Abbreviations

AE: Adverse event
CT: Computed tomography
EBUS: Endobronchial ultrasound
EBUS-TBNA: Endobrochial ultrasound guided transbronchial needle aspiration
ECOG: Eastern cooperative oncology group
EUS: Endoscopic ultrasound
EUS-B: Endoscopic ultrasound using endobronchial ultrasound scope
ICMJE: International Committee of Medical Journal Editors
IRB: Institutional review board
ICH-GCP: International council for harmonisation guidelines for good clinical practice
MRI: Magnetic resonance imaging
NSCLC: Non-small cell lung cancer
OCREB: Ontario cancer research ethics board
PET: Positron emission tomography
ROSE: Rapid onsite cytologic evaluation
SABR: Stereotactic ablative radiotherapy

## References

[1] Ettinger DS, Wood DE, Aisner DL, Akerley W, Bauman J, Chirieac LR (2017). Non-small cell lung Cancer, version 5.2017, NCCN clinical practice guidelines in oncology. J Natl Compr Cancer Netw.

[2] Hellman S, Weichselbaum RR (1995). Oligometastases. J Clin Oncol.

[3] Palma DA, Salama JK, Lo SS, Senan S, Treasure T, Govindan R (2014). The oligometastatic state - separating truth from wishful thinking. Nat Rev Clin Oncol.

[4] Palma DA, Olson R, Harrow S, Gaede S, Louie AV, Haasbeek C (2019). Stereotactic ablative radiotherapy versus standard of care palliative treatment in patients with oligometastatic cancers (SABR-COMET): a randomised, phase 2, open-label trial. Lancet..

[5] Silvestri GA, Gonzalez AV, Jantz MA, Margolis ML, Gould MK, Tanoue LT (2013). Methods for staging non-small cell lung cancer: diagnosis and management of lung cancer, 3rd ed: American College of Chest Physicians evidence-based clinical practice guidelines. Chest..

[6] Fischer B, Lassen U, Mortensen J, Larsen S, Loft A, Bertelsen A (2009). Preoperative staging of lung cancer with combined PET-CT. N Engl J Med.

[7] Schmidt-Hansen M, Baldwin DR, Hasler E, Zamora J, Abraira V, Roqué I, et al. PET-CT for assessing mediastinal lymph node involvement in patients with suspected resectable non-small cell lung cancer. Cochrane Database Syst Rev. 2014;(11):CD009519. 10.1002/14651858.CD009519.pub2. 10.1002/14651858.CD009519.pub2 PMC6472607 25393718

[8] Um S-W, Kim HK, Jung S-H, Han J, Lee KJ, Park HY (2015). Endobronchial ultrasound versus mediastinoscopy for mediastinal nodal staging of non-small-cell lung cancer. J Thorac Oncol.

[9] Ong P, Grosu H, Eapen GA, Rodriguez M, Lazarus D, Ost D (2015). Endobronchial Ultrasound-guided Transbronchial Needle Aspiration for Systematic Nodal Staging of Lung Cancer in Patients with N0 Disease by Computed Tomography and Integrated Positron Emission Tomography–Computed Tomography. Annals ATS.

[10] Vial MR, O’Connell OJ, Grosu HB, Hernandez M, Noor L, Casal RF (2018). Diagnostic performance of endobronchial ultrasound-guided mediastinal lymph node sampling in early stage non-small cell lung cancer: a prospective study. Respirology..

[11] Crombag LMM, Dooms C, Stigt JA, Tournoy KG, Schuurbiers OCJ, Ninaber MK, et al. Systematic and combined endosonographic staging of lung cancer (SCORE study). Eur Respir J. 2019;53(2) [cited 2020 Oct 9]. Available from: https://erj-ersjournals-com.proxy1.lib.uwo.ca/content/53/2/1800800. 10.1183/13993003.00800-2018 30578389

[12] Martinez-Zayas G, Almeida FA, Simoff MJ, Yarmus L, Molina S, Young B (2020). A prediction model to help with oncologic mediastinal evaluation for radiation: HOMER. Am J Respir Crit Care Med.

[13] Tan S, Sharma K, Tham KY, Ang SY, Nguyen VH, Lapperre TS, et al. Comparing performance and cost of EBUS-TBNA versus other methods for diagnosis and staging of non-small cell lung cancer (NSCLC). Eur Respir J. 2014;44(Suppl 58) [cited 2021 Dec 17] Available from: https://erj.ersjournals.com/content/44/Suppl_58/P340.

